# Data sets of migration barriers for atomistic Kinetic Monte Carlo simulations of Fe self-diffusion

**DOI:** 10.1016/j.dib.2018.04.060

**Published:** 2018-04-24

**Authors:** Ekaterina Baibuz, Simon Vigonski, Jyri Lahtinen, Junlei Zhao, Ville Jansson, Vahur Zadin, Flyura Djurabekova

**Affiliations:** a Helsinki Institute of Physics and Department of Physics, University of Helsinki, P.O. Box 43, Pietari Kalmin Katu 2, FI-00014, Finland; b Institute of Technology, University of Tartu, Nooruse 1, 50411 Tartu, Estonia; c National Research Nuclear University MEPhI, Kashirskoye sh. 31, 115409 Moscow, Russia

**Keywords:** Copper, Iron, Kinetic Monte Carlo, Surface diffusion, Rigid lattice, Migration barriers, Atomic jumps

## Abstract

Atomistic rigid lattice Kinetic Monte Carlo (KMC) is an efficient method for simulating nano-objects and surfaces at timescales much longer than those accessible by molecular dynamics. A laborious and non-trivial part of constructing any KMC model is, however, to calculate all migration barriers that are needed to give the probabilities for any atom jump event to occur in the simulations. We calculated three data sets of migration barriers for Fe self-diffusion: barriers of first nearest neighbour jumps, second nearest neighbours hop-on jumps on the Fe {100} surface and a set of barriers of the diagonal exchange processes for various cases of the local atomic environments within the 2nn coordination shell.

**Specifications table**

**Table:** 

Subject area	*Physics*
More specific subject area	*KMC simulations of surface diffusion*
Type of data	*Table*
How data was acquired	*Nudged Elastic band calculations with semi-empirical potentials*
Data format	*raw*
Data source location	*Helsinki, Finland*
Data accessibility	*data is with this article*

**Value of the data**

• *Fe Set 1*, *Fe Set 2NN* and *Fe Set Exchange* tables of migration energy barriers can be used for atomistic rigid lattice Kinetic Monte Carlo simulations of self-diffusion on arbitrarily rough surfaces via first and second nearest neighbour jumps and diagonal exchange processes.
• Bulk diffusion is also possible to simulate with these data sets in KMC.

## 1 Data

*Fe Set 1* and *Fe Set 2NN* tables accompanying this article contain 5 columns named *a, b, c, d, Em,* where *a* and *b* are the numbers of first nearest neighbours (1nn) and second nearest neighbours (2nn) of the initial configuration of the jumping atom, respectively; *c* and *d* - the corresponding numbers for the final vacant lattice site (*c* in *Fe Set 1* and *d* in *Fe Set 2NN* include the jumping atom itself in its initial position); *Em* is the energy barrier in eV, which the jumping atom needs to overcome in order to make a transition from its initial configuration to the final vacant lattice site. The description where the barrier is described by the number of 1nn and 2nn atoms will henceforth be called the 4D parameterization. *Fe Set 1* includes the barriers for 1nn jumps; *Fe Set 2NN –* 2nn hop-on jumps on the Fe {100} surface.

*Fe Set Exchange* includes the barriers of diagonal exchange processes on Fe {100} surfaces for all possible combinations (excluding the symmetric ones) of 2nn atoms of the initial adatom and the final position of the dislodged surface atom. *Fe Set Exchange* has the following format:

s0s1s2s3s4s5Em

where *s0, … s5* are the occupation states (1 = occupied, 0 = vacant) of the six 2nn adatoms’ sites around the initial adatom and the final position of the dislodged surface atom, *Em* is the corresponding barrier value in eV. All sets are described and analysed in detail in [1].

## 2 Computational methods

**Fig. 1:**
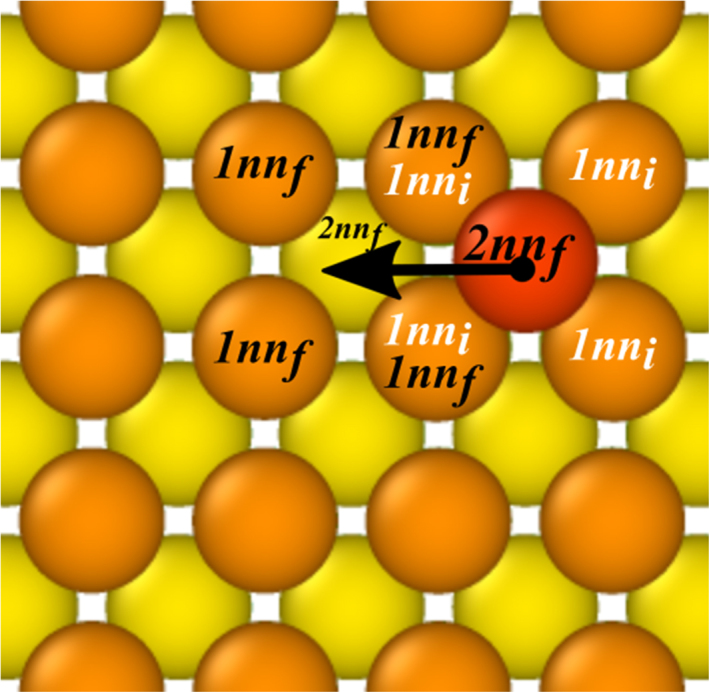
Illustration of a *(4,1,4,2)* 2nn jump on the {100} BCC surface in 4D parameterization scheme. The adatom (red circle) jumps from the site with four 1nn atoms and one 2nn atom (the atom right below the jumping atom) to a site with four 1nn and two 2nn atoms (including the jumping atom itself) within the same monolayer. (For interpretation of the references to color in this figure legend, the reader is referred to the web version of this article.)

*Fe Set 1* and *Fe Set 2NN* were constructed within 4D parameterization scheme of the KMC code Kimocs [2]. In the 4D description of the atomic jumps, only the numbers of 1nn and 2nn of the initial and final sites of the transition are taken into account, but not the precise arrangement of these neighbours (see Fig. 1 for an example of a 2nn jump). Thus, a single value of the energy barrier is assigned to the whole set of various permutations corresponding to the same (a, b,c, d) 4D vector. Such an approach significantly reduces the set of necessary barriers within the 2nn coordination shell from ~$10^{6}$ down to ~2000 for 1nn jumps in body-centred cubic (BCC) lattice structures.

**Fig. 2:**
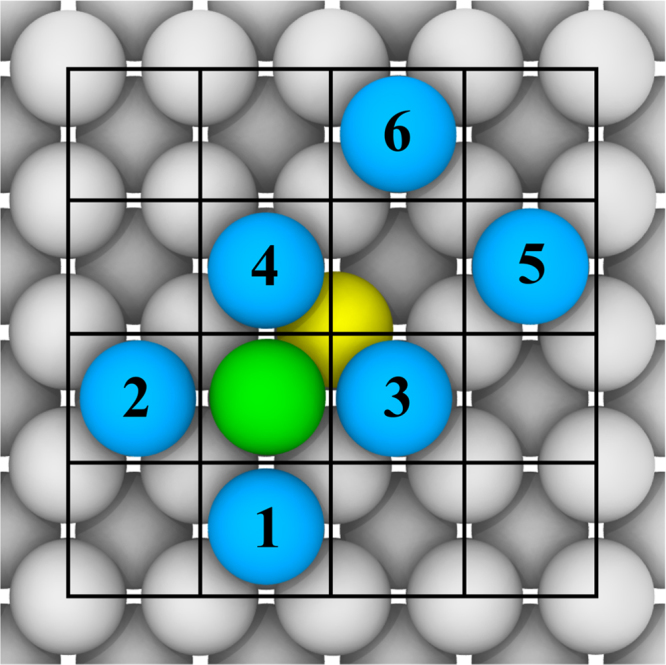
Atoms arrangement in *Fe Set Exchange*. The jumping adatom is shown in green color. The surface atom, which the jumping adatom pushes up is shown in yellow. Blue color corresponds to the occupation site of the second nearest adatoms of the initial green adatom and the final hollow site position of the yellow atom. (For interpretation of the references to color in this figure legend, the reader is referred to the web version of this article.)

*Fe Set Exchange* consists of the barriers of the diagonal exchange processes on Fe {100} surface for all possible combinations of 2nn within the same monolayer as the initial adatom and the final position of the dislodged atom. In Fig. 2, the initial adatom on top of the Fe {100} surface is shown in green. It dislodges the yellow surface atom and takes its place. The yellow atom is forced to become an adatom and takes the diagonal hollow vacant site (between the adatoms 3, 4, 5 and 6). Numbered adatoms are 2nn adatoms of the initial green adatom and a final hollow site position of the yellow atom.

All the barriers in all three sets were calculated using the Nudged Elastic Band (NEB) method [3], [4] with the EAM interatomic potential, developed by Mendelev et al. [5]. The initial and final configurations of every process were constructed in a rigid lattice, either on a surface or in bulk (see [1] for details). During the initial relaxation stage, the initial and final configurations of the processes were relaxed with the conjugate gradient method. The straight line from the relaxed initial position to the relaxed final one was chosen as the initial guess of the minimum energy path (MEP) for 1nn and 2nn jumps. The interpolation path for diagonal exchange processes was chosen to go through the state, in which both the adatom and the surface atom occupy the same lattice site. The minimization of the interpolated path towards the minimum energy path (MEP) was handled by the NEB algorithm in the Molecular Dynamics code PARCAS [6], [7], [8]. We used the approach described in [9] for the calculation of the additional NEB spring force between the images. A sequence of 40 images was used for every jump. The initial and final images were relaxed with the conjugate gradient method and then fixed during the NEB calculations. The energy barrier is found from the relaxed MEP as a difference in the potential energies of the configuration at the saddle point and the initial configuration.

Some processes in *Fe Set 1* were identified as spontaneous during NEB (see [1], [10] for details) and assigned the barriers with the following heuristic formula:

(1) $$E_{m}\left(a,b,c,d\right)=\epsilon a+\delta b+\epsilon c^{-1}+\delta d^{-1}$$

where $\epsilon =10^{-3}$ eV and $\delta =10^{-4}$ eV. This formula is designed to prioritize the jumps of atoms with the fewest neighbouring atoms. It also assumes that it is more favourable for an atom to jump into a position with a higher number of neighbours. ϵ and δ are chosen so that the number of 1nn atoms contributes more into the value of migration barriers than the number of 2nn atoms.

*Fe Set 1* includes 1760 barriers, most of which were calculated in the bulk. Barriers for jumps on the {110} surfaces were calculated separately and are prioritized in this set. 214 barriers were assessed by using Eq. (1). *Fe Set 2NN* contains 16 (a, b, c, d) barriers for the direct jumps of adatoms to a vacant 2nn site on the {100} surface. The permutations for each event in *Fe Set 1* and *Fe Set 2NN* were chosen randomly. *Fe Set 1 and Fe Set 2NN* were combined and used in [11], where the attempt frequency for all events was assumed to be ν_D_ = 6 · 10^12^ s^−1^ [12].

**Fig. 3:**
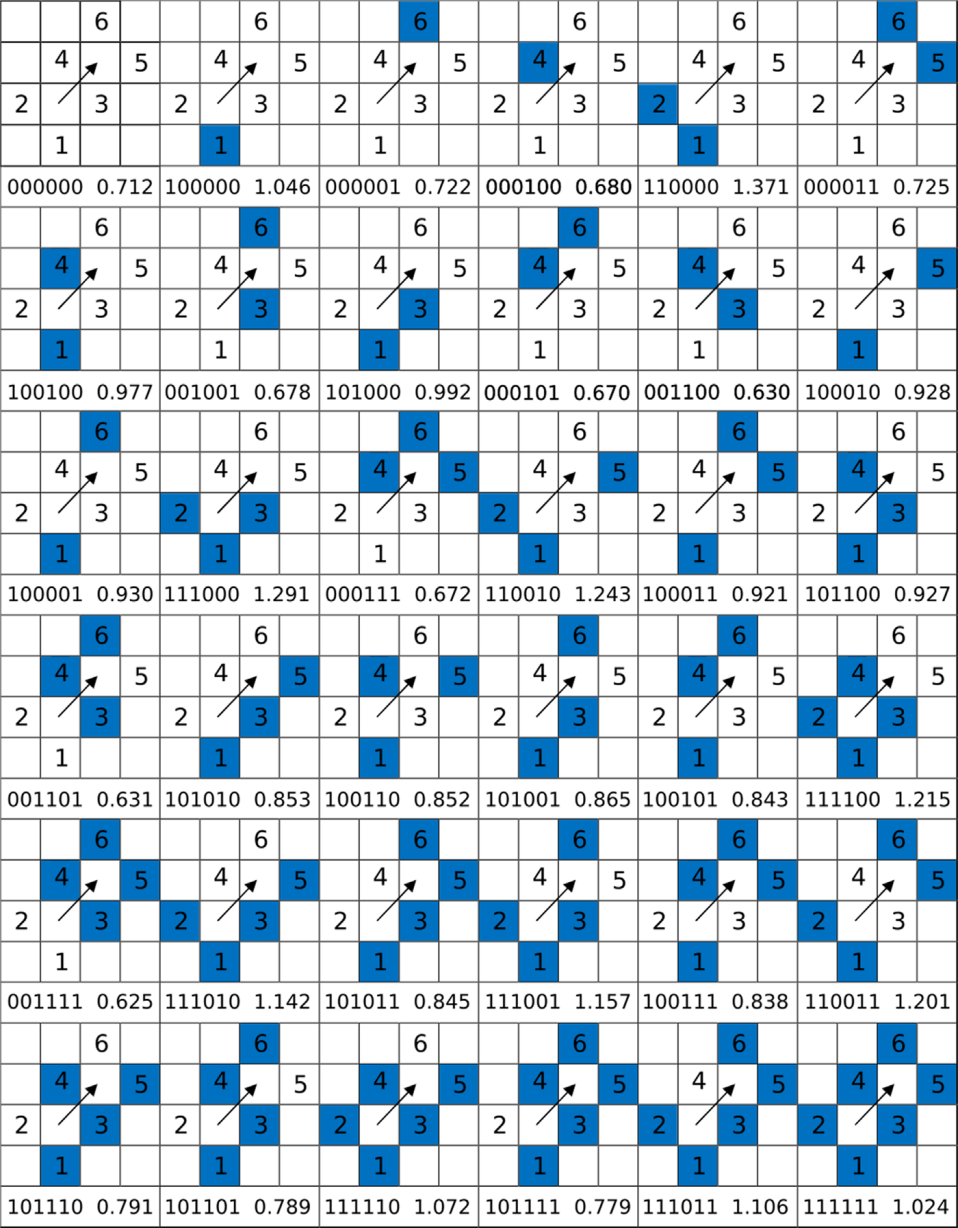
Table of energy barriers of adatom exchange events with various local atomic environments of the jumping adatom and the surface atoms involved in substitution. Processes are described by 6 numbers: *s0 s1 s2 s3 s4 s5 Em*,where *s0, … s5* are the occupation states (1 = occupied, 0 = vacant) of the six 2nn adatom sites around the initial adatom and the final position of the dislodged surface atom, *Em* is the corresponding barrier value in eV. The presented neighbours are shown in blue color.

*Fe Set Exchange* includes 36 barriers. Barriers of various arrangements of numbered 2nn, which are included in *Fe Set Exchange*, are illustrated in Fig. 3 for readers’ convenience. Blue squares mean that the numbered adatom is present, white squares correspond to the situations where 2nn sites are vacant. Fig. 2 correspond to the lower right corner case in the table in Fig. 3.
