# Supplementary material for: Data sets of migration barriers for atomistic Kinetic Monte Carlo simulations of Fe self-diffusion
Source: Data Brief. 2018 Apr 24;19:564–9. doi: 10.1016/j.dib.2018.04.060 (PMC5997586; doi:10.1016/j.dib.2018.04.060)
Supplement: Supplementary file 1 — Supplementary material [file mmc1.docx]

**Conflict of Interest**

The authors have no conflict of interest to declare
